# MiR-519d-3p in Trophoblastic Cells: Effects, Targets and Transfer to Allogeneic Immune Cells via Extracellular Vesicles

**DOI:** 10.3390/ijms21103458

**Published:** 2020-05-14

**Authors:** Wittaya Chaiwangyen, José M. Murrieta-Coxca, Rodolfo R. Favaro, Stella M. Photini, Ruby N. Gutiérrez-Samudio, Ekkehard Schleussner, Udo R. Markert, Diana M. Morales-Prieto

**Affiliations:** 1 Placenta Lab, Department of Obstetrics, Jena University Hospital, 07740 Jena, Germany; wittaya.chaiwangyen@med.uni-jena.de (W.C.); JoseMartin.MurrietaCoxca@med.uni-jena.de (J.M.M.-C.); rodolfo.favaro@med.uni-jena.de (R.R.F.); srayer21@gmail.com (S.M.P.); ruby.gutierrez@med.uni-jena.de (R.N.G.-S.); Ekkehard.Schleussner@med.uni-jena.de (E.S.); diana.morales@med.uni-jena.de (D.M.M.-P.); 2 Division of Biochemistry, School of Medical Sciences, University of Phayao, Phayao 56000, Thailand

**Keywords:** C19MC, cell–cell communication, pregnancy, NK cells, T cells, extracellular vesicles

## Abstract

Members of the placenta-specific miRNA cluster C19MC, including miR-519d, are secreted by fetal trophoblast cells within extracellular vesicles (EVs). Trophoblast-derived EVs can be internalized by the autologous trophoblast and surrounding maternal immune cells, resulting in coordination of cellular responses. The study of functions and targets of placental miRNAs in the donor and recipient cells may contribute to the understanding of the immune tolerance essential in pregnancy. Here, we report that miR-519d-3p levels correlate positively with cell proliferation and negatively with migration in trophoblastic cell lines. Inhibition of miR-519d-3p in JEG-3 cells increases caspase-3 activation and apoptosis. PDCD4 and PTEN are targeted by miR-519d-3p in a cell type-specific manner. Transfection of trophoblastic cell lines with miR-519d mimic results in secretion of EVs containing elevated levels of this miRNA (EV_miR-519d_). Autologous cells enhance their proliferation and decrease their migration ability when treated with EV_miR-519d_. NK92 cells incorporate EV-delivered miR-519d-3p at higher levels than Jurkat T cells. EV_miR-519d_ increases the proliferation of Jurkat T cells but decreases that of NK92 cells. Altogether, miR-519d-3p regulates pivotal trophoblast cell functions, can be transferred horizontally via EVs to maternal immune cells and exerts functions therein. Vesicular miRNA transfer from fetal trophoblasts to maternal immune cells may contribute to the immune tolerance in pregnancy.

## 1. Introduction

Healthy pregnancy relies on the ability of trophoblast cells to proliferate, differentiate and invade the maternal tissues [1,2]. In doing so, trophoblast cells encounter maternal immune cells, such as decidual natural killer (NK) cells, T cells and macrophages [3]. Fetal and maternal cells establish a dialogue that reciprocally controls the recruitment of immune cells to the implantation site, and trophoblast invasion and proliferation [4]. These processes are influenced by genetic, environmental and physiological factors, and their disruption is tightly associated with pregnancy pathologies, including preeclampsia and placenta accreta, intrauterine growth restriction and preterm birth [5]. Compelling evidence indicates that microRNAs (miRNAs) are major regulators of trophoblast and immune cell functions, and thus, influence pregnancy outcomes [6,7,8,9,10].

miRNAs are small (~22 nucleotides) noncoding RNAs that control gene expression at the post-transcriptional level [11,12]. Trophoblast cells express, among others, three major miRNA clusters: the chromosome 19 microRNA cluster (C19MC), C14MC and the miR-371-3 cluster [10,13,14]. Gene orthology analyses show that these clusters are linked to the evolution of the human placenta. C14MC miRNAs are expressed only in placental mammals while C19MC and miR-371-3 miRNAs are restricted to great apes and humans [10,14].

miR-519d-3p belongs to C19MC and constitutes one of the ten highest expressed miRNAs (out of 762 analyzed miRNA) in third trimester trophoblast cells [14]. In pregnancy, miR-519d is highly expressed in maternal blood and decreases significantly within a few days after delivery [15,16]. The function of miR-519d remains widely unclear and is reported to be cell-specific. In several highly invasive human cancers, including hepatocellular and cervical carcinoma, high miR-519d expression has been documented. However, other studies describe that miR-519d attenuates invasion in breast cancer and in trophoblast cells (reviewed in [17]). 

C19MC members including miR-519d-3p are secreted by trophoblast cells via extracellular vesicles (EVs), potentially for communication with neighboring and distant cells [18]. At least two populations of EVs, small (sEVs; 30–120 nm) and large-EVs (lEV; 100–1000 nm), contain miRNAs and can be transferred from trophoblast to immune cells, where they are able to regulate functions [19]. 

In this study, we aimed to investigate the effects of miR-519d-3p on trophoblast cell proliferation and migration and to identify its potential targets. Additionally, the transfer of miR-519d-3p from trophoblast cell lines to different immune cells via EVs and the subsequent effects have been studied. Two trophoblastic cell lines widely used as models for physiological trophoblast cells have been selected as models: JEG-3 derived from choriocarcinoma, and HTR-8/SVneo obtained by immortalization of first trimester trophoblast cells with the gene encoding for simian virus 40 large T antigen [20,21]. These cell lines have marked differences in the expression patterns of genes, proteins and miRNAs, including C19MC species [14,22,23]. Therefore, their simultaneous study facilitates a more reliable extrapolation for the sake of understanding and interpreting molecular processes in primary cells. 

## 2. Results

### 2.1. Expression of miR-519d-3p in Trophoblastic Cell Lines and Their Secreted EVs

**Figure 1:**
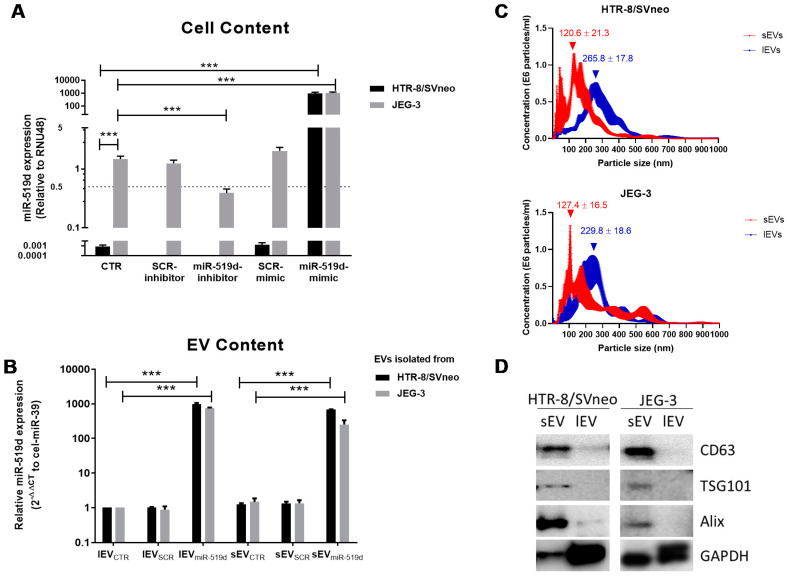
Quantification of miR-519d-3p in cells and EVs. Expression of miR-519d-3p in (**A**) cells and (**B**) their secreted EVs determined by qPCR. Cells were transfected with miR-519d mimic (20 nM), miR-519d inhibitor (120 nM) or the respective scramble sequences SCR mimic and SCR inhibitor. After 48 h, EVs were enriched from cell culture supernatants. Non-detectable levels in HTR8/SVneo cells were defined as Ct 40 for subsequent calculations. miR-519d-3p expression in cells was normalized to RNU48, and in secreted vesicles to spiked cel-miR-39. Data are presented as means ± SDs, *n* = 3. Two-way ANOVA with Bonferroni multiple comparison test; *** *p* < 0.001. (**C**) Nanoparticle tracking analysis (NTA) of sEV (small EV, red line) and lEV (large EV) fractions (blue line) isolated from HTR-8/SVneo (upper) and JEG-3 cell (lower) supernatants. The graph shows EV concentration of depending on size, mean ± SE (*n* = 5). (**D**) Western blotting for EV-associated proteins.

miR-519d-3p was highly and constitutively expressed in JEG-3 cells, but not detectable in HTR-8/SVneo cells (Ct >40). Transfection with miR-519d inhibitor significantly decreased miR-519d-3p expression (~70 % reduction) in JEG-3 cells. miR-519d mimic transfection increased miR-519d-3p expression to similar levels in both JEG-3 and HTR-8/SVneo cells. Due to constitutive expression of this miRNA in JEG-3 cells, transfection promoted a >1200 fold increment on its level, whereas in HTR-8/SVneo cells the values were >40,000 fold higher (Figure 1A). 

Using ultracentrifugation, two populations of enriched EVs were obtained. Following the MISEV2018 guidelines [24], these populations were denotated small or large EVs (sEV or lEV, respectively). EVs enriched from JEG-3 and HTR-8/SVneo cells had similar average sizes (mode ± SE for lEV: 229.8 ± 18.6 vs. 265.8 ± 17.8 nm, and sEV: 127.4 ± 16.5 vs. 120.6 ± 21.3 nm, respectively), and concentrations (×10^6^ particles/mL ± SE for lEV: 1.63 ± 0.17 vs. 1.41 ± 0.08, and sEV: 1.53 ± 0.12 vs. 1.56 ± 0.04, respectively (Figure 1C). CD63, tumor susceptibility gene 101 protein (TSG101) and ALG-2 interacting protein X (ALIX) were enriched in sEV, and barely detected in lEV fractions. Glyceraldehyde-3-phosphate dehydrogenase GAPDH was recovered in sEV and lEV fractions from both cell lines but was more abundant in the lEV fractions (Figure 1D). After transfection of trophoblast cell lines with miR-519d mimic, their sEV and lEV fractions contained significantly more miR-519d: sEV_miR-519d_ (677.2- and 255-fold) and lEV_miR-519d_ (972.8- and 749.3-fold) from HTR-8/SVneo and JEG-3 cells, respectively (Figure 1B).

### 2.2. The Effects of miR-519d-3p on Trophoblast Cell Proliferation and Migration

**Figure 2:**
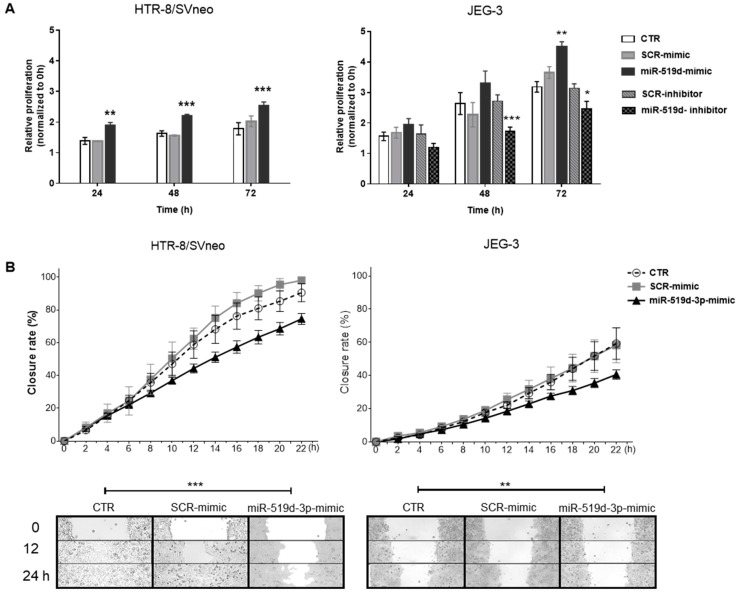
The effect of miR-519d-3p on trophoblastic cell behavior. HTR-8/SVneo and JEG-3 cells were transfected with miR-519d mimic or the scramble sequence SCR mimic for 48 h. As JEG-3 cells express miR-519d, they were additionally transfected with miR-519d inhibitor and SCR inhibitor. Cells were seeded for (**A**) proliferation assay (BrdU incorporation assay) and (**B**) wound healing migration assay. Six areas were photographed (10X) and repopulation was monitored using the JuLI™ Stage cell imaging system. Data are presented as means ± SDs, *n* = 3. Two-way ANOVA with Bonferroni multiple comparison test. * *p* < 0.05, ** *p* < 0.01, *** *p* < 0.001 compared to non-transfected cells (CTR).

Trophoblast cell proliferation and migration are important processes in the establishment and maintenance of healthy pregnancy. To evaluate its roles in these processes, miR-519d-3p was overexpressed in both cell lines and inhibited in JEG-3 cells. Upon overexpression of miR-519d, proliferation increased significantly in both cell lines beginning at 24h in HTR-8/SVneo and at 72 h in JEG-3 cells. Inhibition of miR-519d-3p significantly decreased JEG-3 cell proliferation at 48–72 h (Figure 2A). JEG-3 cells proliferated more but migrated less than HTR8-SVneo cells. miR-519d-3p had a negative effect on trophoblast cell migration, as assessed through a wound healing migration assay. In both trophoblastic cell lines, transfection with miR-519d mimic significantly decreased migration compared to non-transfected cells or transfected with a non-genomic scramble sequence (SCR mimic; Figure 2B).

### 2.3. The Effect of miR-519d-3p Inhibition on the Apoptosis of Trophoblastic Cells

**Figure 3:**
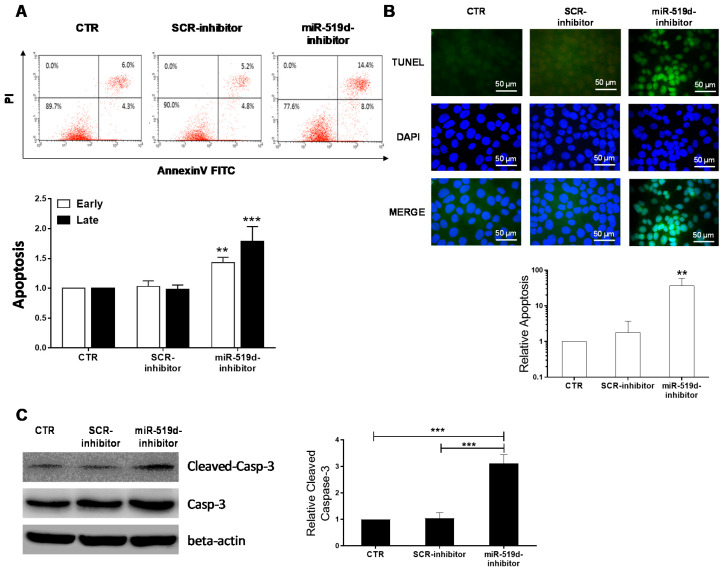
Induction of apoptosis by miR-519d-inhibition. (**A**) Annexin V staining detected by FACS. JEG-3 cells were labelled with Annexin V-FITC and PI 48 h after transfection with miR-519d inhibitor or the respective scramble sequences. Upper: exemplary flow cytometry analysis. Lower: Statistical analysis of early and late apoptotic cells (*n* = 3). Bars show the mean + SD. (**B**) Upper: TUNEL assay indicating apoptotic cells (green) and nuclei counterstained with DAPI (blue). Scale bars: 50 μm. Lower: Mean ratio of apoptotic cell number in treated cells to that in controls. Error bars indicate SD. (**C**) Caspase-3 expression in transfected JEG-3 cells. Bars represent means + SDs, *n* = 3. Two-way ANOVA with Bonferroni multiple comparison test (**A**) and two-tailed *t*-test (**B**,**C**). ** *p* < 0.01, *** *p* < 0.001.

The decrease observed in cell viability after miR-519d-3p inhibition may be associated with an increased apoptosis rate. To further evaluate this hypothesis, apoptosis was assessed by Annexin V staining and TUNEL assay in JEG-3 cells after transfection with miR-519d-3p inhibitor. As HTR/SVneo cells lack miR-519d-3p expression, they were not used for this assay. Early (Annexin V+/PI-) and late apoptotic cells (Annexin V+/PI+) were augmented significantly of the JEG-3 cells transfected with miR-519d inhibitor, compared to controls (Figure 3A). Apoptotic DNA cleavage was also elevated in JEG-3 cells after miR-519d-3p inhibition, as assessed by TUNEL assay (Figure 3B). DNA fragmentation and degradation of cytoskeletal proteins are often associated with activation of caspase-3, a member of the family of endoproteases. Therefore, cleaved caspase-3 is considered an indicator of progressing apoptosis. In our hands, inhibition of miR-519d-3p in JEG-3 cells also resulted in increased cleaved caspase-3 detected by Western blotting (Figure 3C). Altogether, the data demonstrate that miR-519d-3p inhibition induces cell apoptosis via caspase-3 activation.

### 2.4. Targets of miR-519d-3p in Trophoblastic Cell Lines

**Figure 4:**
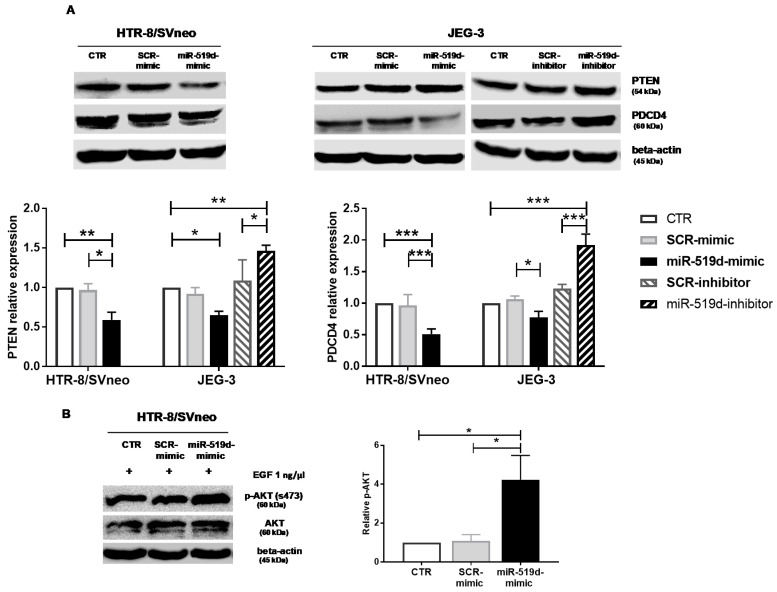
miR-519d-3p targets PTEN and PDCD4 in trophoblastic cell lines. HTR-8/SVneo and JEG-3 cells were transfected with miR-519d mimic or the respective scramble sequence, SCR mimic. Additionally, JEG-3 cells were transfected with inhibitors of miR-519d-3p and the respective control, SCR inhibitor. After 48 h, Western blots and densitometric analyses were carried out for (**A**) PTEN (left) and PDCD4 (right). (**B**) p-AKT and AKT have been assessed upon further stimulation with EGF (1 ng/µL, for 5 min) in HTR-8/SVneo. Bars represent means ± SDs, *n* = 3 (PTEN and AKT) and *n* = 4 (PDCD4). Two-way (PTEN and PDCD4) and one-way ANOVA (AKT). * *p* < 0.05, ** *p* < 0.01, *** *p* < 0.001.

A bioinformatic analysis of genes involved in cell proliferation, invasion and apoptosis identified PTEN and PDCD4 as putative miR-519d-3p targets. As assessed by Western blotting, mRNA targets of miR-519d-3p were cell-type-specific. PDCD4 was upregulated in JEG-3 cells after transfection with miR-519d inhibitor and downregulated after transfection with miR-519d mimic. In HTR8/SVneo cells, PDCD4 levels were unaltered (Figure 4A right). Transfection with miR-519d mimic significantly reduced PTEN in HTR-8/SVneo, but not in JEG-3 cells (Figure 4A left). The PTEN/PI3K/AKT system constitutes an important signaling pathway of cell proliferation and metabolism, and thus, activation of AKT was investigated in HTR-8/SVneo cells where miR-519d-3p targets PTEN. To measure AKT activation, cells were treated with epithelial growth factor (EGF) after transfection with miR-519d mimics. Increased EGF-induced AKT phosphorylation was observed in transfected cells, confirming the capacity of miR-519d-3p in regulating the PTEN/AKT pathway in HTR-8/SVneo cells (Figure 4B).

### 2.5. Uptake of EVs by Autologous Trophoblast Cells

**Figure 5:**
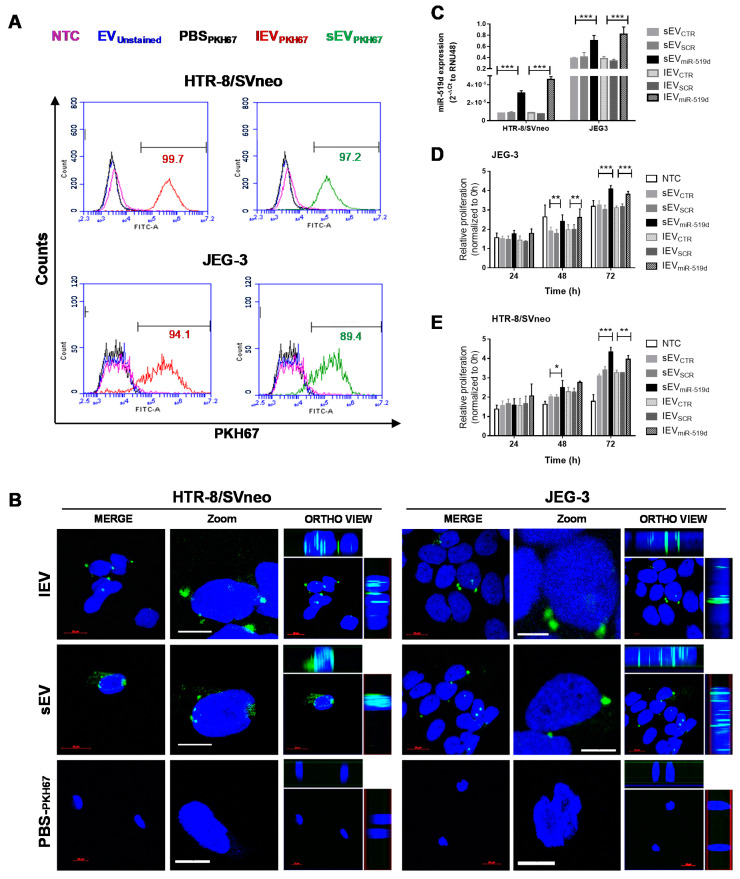
Uptake of trophoblastic EVs by autologous cells. (**A**) Flow cytometry analysis of HTR-8/SVneo and JEG-3 cells co-incubated with unstained (EV_unstained_) or PKH-67-labeled EVs (sEV_PKH67_ and lEV_PKH67_) or treated with EV-free PBS_PKH67_. Non-treated cells (NTC). Numbers (green/red) indicate the respective percentages of labeled cells. (**B**) Cellular uptake of autologous EVs (lEV_PKH67_ and sEV_PKH67_) in HTR-8/SVneo and JEG-3 cells imaged by confocal laser scanning microscopy. The fluorescence of DAPI and that of PKH-67 are labeled with blue and green, respectively. Merged are shown for HTR-8/SVneo and JEG-3 cells and displayed in two different magnifications. Ortho-view images of the z-stack show EVs inside the cells. Scale bars: 10 μm. (**C**) miR-519d-3p expression in recipient cells by qPCR normalized to RNU48. (**D**) Cell proliferation of HTR8/SVneo and (**E**) JEG-3 cells relative to NTC at 0 h. **C**–**E**: HTR-8/SVneo and JEG-3 cells were treated with sEVs and lEVs isolated from cells transfected with miR-519d-3p mimic (sEV_miR-519d_ and lEV_miR-519d_), SCR mimic (sEV_SCR_ and lEV_SCR_) or non-transfected controls (sEV_CTR_ and lEV_CTR_). Bars represent means ± SDs, *n* = 3. Two-way ANOVA with Bonferroni multiple comparison test. * *p* < 0.05, ** *p* < 0.01, *** *p* < 0.001.

As shown above, miR-519d-3p regulates the behavior and gene expression of trophoblast cells. Transfer of miR-519d-3p via EVs may serve as a mechanism of synchronization among autologous cells, and therefore, was studied next. Non-transfected JEG-3 and HTR-8/SVneo cells were incubated with sEV or lEV, stained with PKH67 (sEV_PKH67_ and lEV_PKH67_, respectively) or PBS_PKH67_ (control). After 24 h of incubation with sEV_PKH67_ and lEV_PKH67_ but not with PBS_PKH_, a high percentage (>89%) of cells became positive for PKH67, as measured by flow cytometry (Figure 5A). Furthermore, cells incubated with sEV_PKH67_, lEV_PKH67_ or PBS_PKH67_ for 24 h were visualized by confocal microscopy. A merged image is presented showing cellular uptake of trophoblast-derived EVs (lEV_PKH67_ and sEV_PKH67_) in HTR-8/SVneo and JEG-3 cells. Ortho-view images of the z-stack show PKH67 (lEV_PKH67_ or sEV_PKH67_) and DAPI (nuclei) signals colocalizing at the same plane, demonstrating EVs inside the cells (Figure 5B). 3D visualization of internalized EVs demonstrated the localization of lEV and sEV mostly in the perinuclear region (Figures S1–S6). In both cell lines, treatment with 125 ng/mL sEV_miR-519d_ and lEV_miR-519d_ resulted in elevation of intracellular miR-519d-3p level compared to controls (Figure 5C). Compared to non-treated controls, HTR8/SVneo cells treated with sEV_CTR_ and lEV_CTR_ have a higher proliferation rate. This effect did not occur in JEG-3 cells. JEG-3 and HTR-8/SVneo cells proliferated significantly more after treatment with sEV_miR-519d_ and lEV_miR-519d_ compared to controls, and the effect was more pronounced in HTR-8/SVneo cells (Figure 5D,E). 

**Figure 6:**
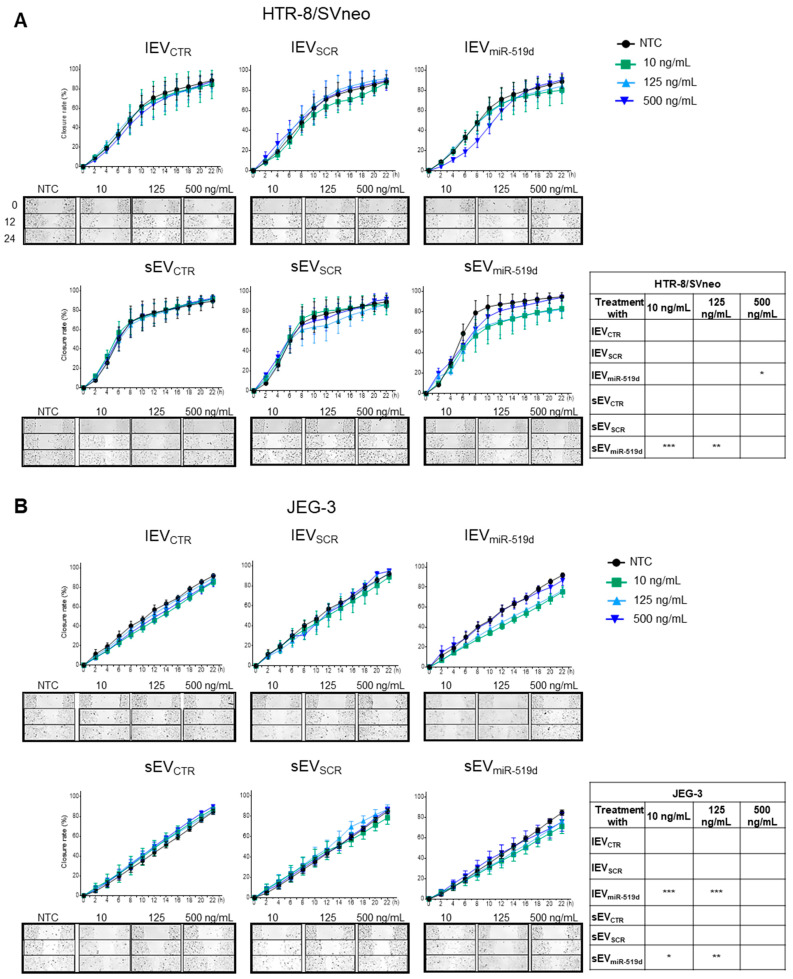
The effect of EVs containing miR-519d-3p on trophoblastic cell migration. (**A**) HTR-8/SVneo and (**B**) JEG-3 cells were treated with sEV and lEV isolated from cells transfected with miR-519d-3p mimic (sEV_miR-519d_ and lEV_miR-519d_), SCR mimic (sEV_SCR_ and lEV_SCR_) or non-transfected controls (sEV_CTR_ and lEV_CTR_) and compared to non-treated cells (NTC). Migration was assessed by using a wound healing migration assay on a JuLI™ Stage automated cell imaging system. Data are presented as means ± SDs, *n* = 3. Two-way ANOVA with Bonferroni multiple comparison test. * *p* < 0.05, ** *p* < 0.01, *** *p* < 0.001 compared to NTC.

A significant reduction in migration was observed in HTR-8/SVneo cells treated with 500 ng/mL of lEV_miR-519d_ and with 10 and 125 ng/mL of sEV_miR-519d_ (Figure 6A). Likewise, JEG-3 cells showed a decreased migration when treated with 10 and 125 ng/mL of sEV_miR-519d_ or lEV_miR-519d_ (Figure 6B). No effects were observed under treatment with EV from non-transfected cells (sEV_CTR_ and lEV_CTR_) or transfected with non-genomic scramble sequences (sEV_SCR_ and lEV_SCR_) at any tested concentration (Figure 6).

### 2.6. The Uptake by Heterologous Immune Cells of EVs, and Their Effects

Trophoblast-derived EV-mediated delivery of C19MC into maternal immune cells occurs in pregnancy and may be assumed to contribute to the maternal tolerance required for a physiological pregnancy. To evaluate the transfer of miR-519d-3p via EVs from trophoblast to immune cells and their function therein in vitro, naïve Jurkat T and NK92 cells were incubated with EVs secreted from JEG-3 and HTR-8/SVneo cells. 

**Figure 7:**
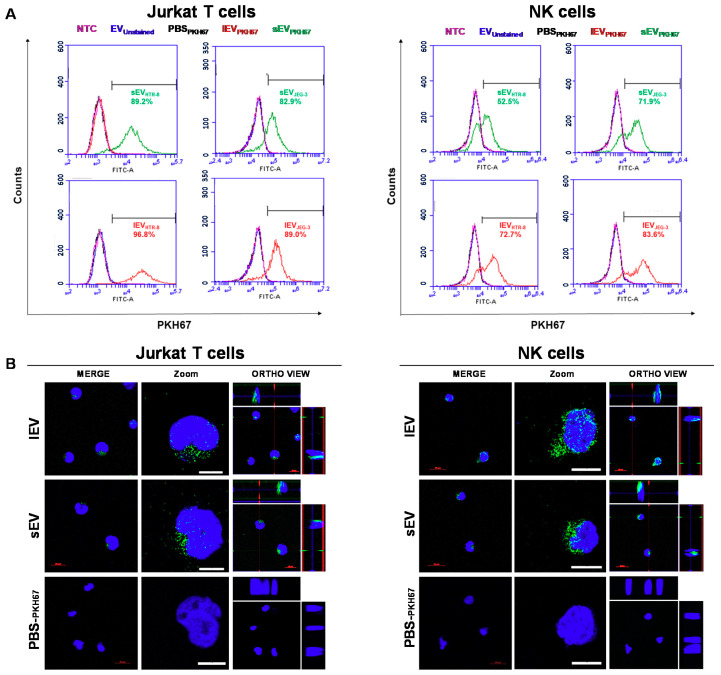

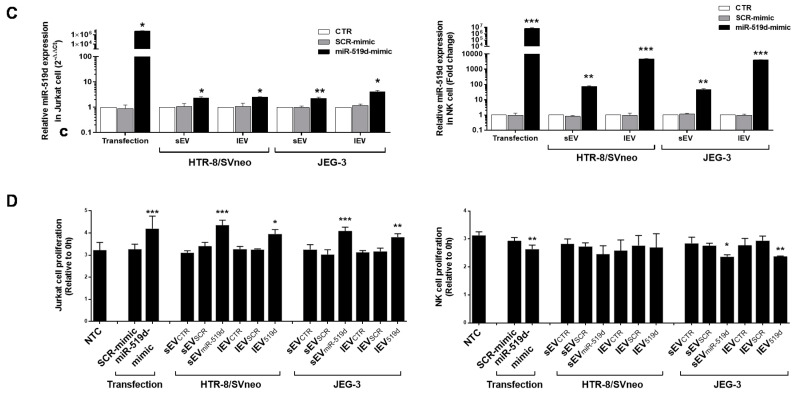
Uptake of trophoblastic EVs by heterologous immune cells. Immune cells were co-incubated with 125 ng/mL unstained (EV_unstained_) or PKH-67-labeled EVs (sEV_PKH67_ and lEV_PKH67_) or treated with EV-free PBS_PKH67_. NTC: non-treated cells. (**A**) Assessment of trophoblastic EV (EV_JEG-3_ or EV_HTR-8/SVneo_) incorporation in immune cells by flow cytometry. The numbers (green/red) indicate the respective percentages of labeled cells. (**B**) Representative cellular uptake of HTR-8/SVneo-derived EVs (lEV_PKH67_ and sEV_PKH67_) in Jurkat T (left) and NK92 (right) cells imaged by confocal laser scanning microscopy. The fluorescence of DAPI and that of PKH-67 are pseudo-labeled with blue and green, respectively, and displayed in two different magnifications. Ortho-view images of z-stack show EVs inside the cells. Scale bars: 10 μm. (**C**) Jurkat T and NK92 cells were transfected directly or treated with EVs enriched from medium conditioned by trophoblastic cell lines transfected with miR-519d mimic or SCR mimic. miR-519d-3p expression in recipient cells by qPCR normalized to RNU48. (**D**) Cell proliferation assessed by BrdU assay at 72 h. Bars represent means ± SDs, *n* = 3. Two-way ANOVA with Bonferroni multiple comparison test. * *p* < 0.05, ** *p* < 0.01, *** *p* < 0.001.

Upon incubation with PKH67-stained trophoblastic EVs (sEV_PKH67_ and lEV_PKH67_) for 24 h, a high percentage of immune cells became positive for PKH67, as assessed by flow cytometry (Figure 7A). No fluorescence shift was observed when cells were incubated with unstained EVs (EV_unstained_) or PKH67-treated PBS (PBS_PKH67_). In NK92 cells, two subpopulations were observed after EV treatment as result of higher or lower-uptake (Figure 7A). Additionally, uptake of PKH67-stained vesicles was visualized by confocal microscopy in Jurkat T (left) and NK92 (right) cells incubated with sEV_PKH67_, lEV_PKH67_ or PBS_PKH67_ for 24 h. Increased uptake in NK92 cells compared to Jurkat T cells was observed by increased green fluorescence. Serial slices were acquired to produce a Z-stack confocal image, which demonstrates internalization and localization of labeled EVs in the cytoplasm and perinuclear regions of target cells (Figure 7B and Figures S7–S12).

Expression of miR-519d-3p was significantly increased in NK92 and Jurkat T cells upon direct transfection with miR-519d mimic or upon treatment with 125 ng/mL sEV_miR-519d_ or lEV_miR-519d_ compared to controls (Figure 7C). Higher changes were observed in NK92 cells compared to Jurkat T cells (Figure 7C). These results were independent of the EV origin (JEG-3 or HTR-8/SVneo cells). 

Treatment with sEV_miR-519d_ or lEV_miR-519d,_ and direct transfection with miR-519d mimic resulted in increased proliferation in Jurkat T cells (Figure 7D left). In contrast, in NK92 cells, transfection with miR-519d mimic and treatment with sEV_miR-519d_ or lEV_miR-519d_ from JEG-3 but not from HTR-8/SVneo cells reduced proliferation (Figure 7D right).

## 3. Discussion

Placental miRNAs are commonly organized in families or clusters which may share regulatory mechanisms and functions [13]. C19MC, the largest human miRNA cluster, is a pregnancy-associated cluster located on chromosome 19q13.42. Despite being found in cancer cells [25], physiological expression of C19MC members is restricted to placental-tissue [26] and increases with gestational age [14,18]. Recent evidence has suggested a pivotal role of C19MC miRNAs in controlling placental development and trophoblast behavior [27,28]. Furthermore, C19MC members can be packed and secreted by placental cells in EVs as a mechanism of communication between neighboring or distant cells [29,30]. Nevertheless, the functions of these miRNAs remain largely unknown.

In this study, we have investigated the function and targets of miR-519d-3p, a C19MC member, in trophoblastic cell lines and its transfer to autologous trophoblastic and heterologous immune cells via EVs. Overexpression of miR-519d has been found in several invasive human cancers, including hepatocellular carcinoma, gastric cancer and breast cancer [31,32]. This potential association with invasiveness may go in line with some reports of placental tissue showing miR-519d down-regulation in pathologies associated with low invasion, such as gestational hypertension, fetal growth restriction and preeclampsia [15,33,34]. Recent studies on extravillous trophoblast cells, however, reported the role of miR-519d and other C19MC members in attenuating cell migration but not proliferation [35]. In our hands, miR-519d-3p expression correlated positively with cell proliferation and negatively with migration capacity in trophoblast cells independently of their basal expression levels. Furthermore, treatment of JEG-3 cells with a specific miR-519d-3p hairpin inhibitor resulted in increased cellular apoptosis indicated by caspase-3 activation, in agreement with recent publications describing this molecular mechanism in cancer cells [36,37]. As expectable, because of their malignant origin, in our experiments JEG-3 cells had a higher proliferative rate than HTR8/SVneo cells. Moreover, JEG-3 choriocarcinoma cells have a constitutively high miR-519d-3p expression, whilst HTR8/SVneo cells, established by immortalization of first trimester trophoblast cells, lack its expression [14,18,28]. The similar response observed in proliferation and migration reported here in two trophoblast cell models demonstrates conserved functions of miR-519d-3p which may be relevant *in vivo.* Recently, we reported differences in the miRNA response of trophoblastic cell lines to leukemia inhibitory factor (LIF) [38]. Therefore, it may be expected that additional stimuli at the materno-fetal interface, such as growth factors or cytokines, may influence the function of miR-519d-3p. Further experiments are required to investigate this assumption. 

As we reported before [19], transfection of trophoblastic cell lines with miR-mimics (double-stranded miRNA-like fragments) results in significant enhancement of specific miRNA levels in both the transfected cells and their secreted EVs. Two populations of EVs were isolated: sEV and lEVs. Despite presenting overlay in their size distribution and inter-experimental differences by nanoparticle tracking analysis (NTA), the size mode of sEV fractions was clearly smaller than that of lEV fractions. Particle size overestimation may have occurred caused by measurement of the larger hydroscopic diameter [39], but aggregate formation can also play a role even, after filtration through 200 nm pore filters. In our hands, biochemical analysis of EV subpopulations confirmed a high degree of separation between fractions. sEVs harbored CD63, ALIX, and TSG101, which were almost undetectable in the lEV fractions. The tetraspanin CD63 localizes predominantly to late endosomes and lysosomes and plays an important role in sorting intraluminal vesicles [40]. ALIX and TSG101 are proteins associated with the endosomal sorting complex required for transport (ESCRT) machinery responsible for sorting cargo into endosome membranes [41]. As a result, CD63, ALIX and TSG101 are considered markers for endosomal-derived EVs. Conversely, GAPDH is a cytosolic protein incorporated in different EV populations [24]. In our hands, it was expressed by both EV fractions. These results together with the size measurements point out to an enrichment of vesicles of endosomal origin in the sEV fractions.

Treatment of trophoblastic cell lines with EVs containing elevated miR-519d-3p levels resulted in a reduction of cell migration. Remarkably, this effect was generally higher at lower EV concentrations, and in HTR-8/SVneo cells, it differs between sEV and lEV. In our setting, it is unfeasible to assess the effect of miR-519d-3p transfer independently of the overall EV effect. The similar effect caused by direct transfection with miR-519d-3p-mimic demonstrates that trophoblast cell lines horizontally transfer miR-519d-3p among autologous cells via EVs, and that it remains functional in the recipient cells, reducing their migration potential. The differences observed at higher concentrations may be caused also by the effect of miR-519d-3p on cell proliferation potentially overlying the effect on migration. Furthermore, other molecules (including additional miRNAs) are transferred during EV treatment and can differ between EV subpopulations [42], which may imply additional mechanisms that counteract the solely the effect of vesicular miR-519d-3p. Further studies are needed to evaluate these mechanisms and the implications for miR-519d-3p transfer in the cellular synchronization of trophoblast cells in physiological situations and in pregnancy pathologies. 

To better elucidate the translational potential of our findings for the in vivo situation, we identified miR-519d-3p targets in trophoblast cells. PTEN and PDCD4 were recognized as putative miR-519d-3p targets in different bioinformatics platforms and were selected because they regulate cell growth, proliferation, migration, invasion and apoptosis in several cell types [24,25,26,27,28]. Similarly to our previous report [9], mRNA targets of miRNAs were found to be cell-type specific. Only in HTR-8/SVneo cells, PTEN was confirmed to be a target of miR-519d-3p. This impacts the associated pathway, as demonstrated in cells treated with miR-519d mimic, which showed higher AKT activation when stimulated with EGF. On the other hand, PDCD4 was confirmed to be a miR-519d-3p target only in JEG-3 cells, supporting its role in controlling apoptosis reported above. This cell-type specificity of miR-519d-3p targets may be due to dissimilarities in the expression of miRNAs and their target mRNAs in trophoblast cell lines [14,22]. Furthermore, regulation of mRNA targets may be time or differentiation-dependent, as occurs in primary trophoblast cells that increasingly express miR-519d-3p throughout pregnancy [14]. Further experiments may clarify the targets of miR-519d-3p in different trophoblast populations. 

Trophoblast cells also communicate with surrounding immune cells either directly by cell–cell contact or indirectly through extracellular mediators [43,44]. During early pregnancy, this process is of outmost importance because trophoblast cells modulate immune cell recruitment, T cell activation and NK cell cytotoxicity at the maternal–fetal interface [45,46,47,48]. Our results demonstrate that T and NK cells bind and incorporate trophoblast-secreted EVs. Assessment of miR-519d-3p levels in the cytoplasm of recipient cells via qPCR suggested a higher trophoblast-derived EV incorporation in NK92 compared to Jurkat T cells. miR-519d-3p levels increased more than 100-fold in NK92 cells treated with EV_miR-519d_ compared to approximately 5-fold in Jurkat T cells. Nevertheless, the behavior of NK92 cells was not homogenous and resulted in two subpopulations characterized by higher and lower EV uptakes. The diverse cytotoxic, metabolic and uptake capacities of specific NK92 subpopulations may be responsible for these observations. The specificity of EV uptake by different NK subpopulations may be relevant for the immunosuppression and tolerance in pregnancy, and thus, is worthy of being investigated. Further studies on other types of EVs are necessary to determine whether NK cells have a generally higher capacity of EV internalization than T cells or this is specific to trophoblast-derived EVs [49]. 

In recipient immune cells, internalized miR-519d-3p remained active and modified their proliferation in a cell-specific manner. EV_miR-519d_ uptake resulted in increased Jurkat T but decreased NK92 cell proliferation. The observed differences were the result of miR-519-3pd internalization rather than a general response to EVs, as confirmed by direct transfection of cells with miR-519d-3p-mimic. Our observations also demonstrate that EV_JEG-3_ were more effective in reducing NK92 proliferation than EV_HTR-8/SVneo_. These differences may rely on their different sizes and molecular compositions, such as the expression of miRNAs, including further members of C19MC [14,18]. The effects on Jurkat T cells induced by miR-519d-3p are opposite to those induced by miR-141, another pregnancy-associated miRNA, which reduces T cell proliferation in the same experimental design [19], demonstrating a miR-specific cell response. 

Cumulating evidence demonstrates the effects of trophoblast-derived EVs on maternal cells starting at the preimplantation period. Embryo-secreted EVs are taken up by the luminal epithelium but not the stroma or myometrium, and their miRNA content is associated with the implantation potential (reviewed by [50]). Furthermore, trophoblast-derived EV cargo includes immunomodulatory factors such as trophoblast glycoprotein, Fas ligand, TRAIL, the non-classical human leucocyte antigen (HLA)-G and progesterone-induced blocking factor (PIBF) whose transfer to immune cells are associated with successful vascular remodeling and pregnancy maintenance ([51,52,53] and reviewed in [54,55]). Trophoblast cells communicate via EVs with maternal immune cells, including B, T and NK cells but the study of their effects remains incipient. In peripheral blood mononuclear cells (PBMCs), lEV induces the release of proinflammatory cytokines, including tumor necrosis factor (TNF), IL-18, IL-12 and IFN-γ; the sEV fraction suppresses PBMC activation mediating the Th1–Th2 cytokine shift [54,55]. Besides the effects on cell proliferation reported in this study, it may be hypothesized that miR-519d-3p-containing EVs also modulate the cytokine profiles of maternal immune cells, thereby contributing to embryo implantation and peripheral tolerance. 

Since one miRNA potentially can target thousands of mRNAs, the selection depends greatly on the simultaneous presence of both the miRNA and the mRNA target within a specific cell. Because immune cells do not constitutively express placenta-specific miRNAs, such as miR-519d-3p, additional regulatory processes may occur in the presence of trophoblast EVs. However, the functionality of this process in vivo has to be confirmed. 

In conclusion, miR-519d-3p regulates trophoblastic cell proliferation and migration. It can be transferred via EVs to autologous trophoblastic or allogeneic immune cells where it is internalized and may regulate cellular processes necessary for adaption of the maternal organism to pregnancy.

## 4. Materials and Methods

### 4.1. Cell Lines and Cell Culture 

The immortalized human trophoblast cell line HTR-8/SVneo (kind gift from CH Graham, Kingston, Canada), the human T lymphocyte cell line Jurkat (ACC 282; DSMZ) and the human natural killer lymphoma cell line NK92 (ACC 488; DSMZ) were cultured in RMPI-1640 medium (PAA laboratories, Pasching, Austria). The choriocarcinoma cell line JEG-3 (DSMZ, Braunschweig, Germany) was cultured in Ham F-12 medium (PAA laboratories, Pasching, Austria). Media were supplemented with 10% heat-inactivated fetal bovine serum (FBS; Sigma, Germany), 50 U/mL penicillin and 50 µg/mL streptomycin (PAA laboratories, Pasching, Austria). Cells were maintained under standardized conditions (37 °C, 5% CO_2_, humidified atmosphere), authenticated by STR DNA profiling analysis and regularly screened for absence of mycoplasma.

### 4.2. Transfection with miR-519d Mimic and miR-519d Inhibitor

Cells were seeded in 6-well plates and allowed to attach overnight to reach 30%–50% confluence at the time of transfection. Transfection was performed for 48 h using Oligofectamine (Invitrogen Life Technologies, Darmstadt, Germany), miRNA mimics (20 nM) and miRNA inhibitors (120 nM) according to the manufacturer’s instructions. miR-519d-3p-inhibitor (IH-300812-06), miR-519d-3p-mimic (C-300812-05) and the respective negative scramble controls, SCR inhibitor (IN-001005-01-05) and SCR mimic (CN-001000-01-05), were obtained from Thermo Fisher Scientific Dharmacon (Schwerte, Germany). Incubation was carried out in media supplemented with exosome-depleted FBS (ED-FBS; Thermo Fisher).

### 4.3. Enrichment of sEV or lEV Populations from Cell Line Supernatants and Protein Quantification

Cells were kept in medium containing 10% ED-FBS for 48 h. EVs were enriched from supernatants by differential centrifugation as previously described [19]. Pellets containing sEV or lEVs were resuspended either in PBS for functional assays or in TRIzol for RNA extraction. Protein equivalents of sEV and lEV fractions were quantified using Micro BCA assay (Pierce™ BCA Protein Assay Kit, Sigma Aldrich, Poole, UK) following the manufacturer’s instructions. Briefly, 150 μL of sample was incubated with 150 μL of the working reagent at 37 °C. After 2 h, absorbance was measured at 562 nm using a SPECTROstar microplate reader. EV concentrations used for further experiments were determined as protein equivalents based on a standard curve of BSA.

### 4.4. Nanoparticle Tracking Analysis (NTA)

Sizes and concentrations of EV suspensions were assessed by NTA on a NanoSight version 2.3 (NanoSight Ltd., Amesbury, UK) as described before [19]. 

### 4.5. Quantification of miR-519d-3p by qPCR 

Total RNA was extracted from cells or EV fractions using TRIzol reagent (Invitrogen life technologies, Damstadt, Germany) according to the manufacturer’s instructions. Total RNA concentration was determined at a NanoDrop ND-1000 spectrophotometer (Thermo Fisher Scientific, Wilmington, DE, USA). Samples with a A260/A280 ratio greater than 1.8 were stored at − 80°C until being processed. 

Expression of miR-519d-3p was measured by qPCR using a TaqMan miRNAs reverse transcription kit (Applied Biosystems, Damstadt, Germany) in a 7300 Real-time PCR System (Applied Biosystems, Damstadt, Germany). For analysis of EV, the *Caenorhabditis elegans* miRNA cel-miR-39 (ID # MSY0000010. 5′-UCACCGGGUGUAAAUCAGCUUG) was added at a concentration of 1.6 × 10^8^ copies/µl and used as spike-in control. Specific TaqMan microRNA assays for hsa-miR-519d-3p (Assay ID: 002403), cel-miR-39 (Assay ID: 000200) and RNU-48 (Assay ID: 001006) were employed. Expression of miR-519d-3p was normalized using the 2^−ΔΔCt^ method relative to RNU48 or cel-miR-39 in cells or EV, respectively. 

### 4.6. Proliferation Assay 

Proliferation was assessed using a colorimetric BrdU-incorporation ELISA kit (Roche Applied Science, Mannheim, Germany) following the manufacturer’s instructions. Cells were seeded in 96 well plates at a density of 5 × 10^3^ cells/well in 200 µL medium containing 10% ED-FBS. lEV or sEV fractions were added at a concentration of 125 ng/mL and cells were cultured for up to 72 h. Subsequently, BrdU-incorporation was completed in 2 h. 

### 4.7. Wound Healing Migration Assay

Cell migration was assessed using Ibidi™ culture inserts (Ibidi, Regensburg, Germany). Briefly, inserts consisting of two chambers, each one with an area of 0.22 cm^2^ and separated by a 0.5 mm divider, were placed on the well surfaces of 24-well plates. A cell suspension of 1 × 10^6^ cells/mL was prepared and 70 µL was transferred to each chamber. After cell attachment, culture inserts were gently removed, and wells were filled with 1 mL of supplemented media (10 % ED-FBS and 1% streptomycin-penicillin) containing different concentrations (10, 125 and 500 ng/mL) of lEV or sEV fractions. Wound closure was monitored every hour during a period of 24 h using a JuLI™ Stage automated cell imaging system (NanoEnTek, Seoul, Korea). 

### 4.8. Apoptosis Assays

Cells were harvested and washed twice in cold PBS. Thereafter, cells were stained with FITC annexin V and propidium iodide (PI; both Immunotools, Friesoythe, Germany) for 15 min and then measured at a FACS Calibur and analyzed by using Cell Quest software (both Becton Dickinson Co., Franklin Lakes, NJ, USA). Percentage of early and late apoptotic cells was determined in 10,000 events. Annexin V-positive/PI-negative cells were designated as early and Annexin V-positive/PI-positive cells as late apoptotic. 

Apoptosis was additionally evaluated using The DeadEnd™ Fluorometric TUNEL System (Promega, Walldorf, Germany), according to the manufacturer’s instructions. Cells were cultured on slides at a total number of 250,000. After 24 h, they were fixed in 4% methanol-free formaldehyde and permeabilized with Triton^®^ X-100. DNA strand breaks were labeled with fluorescein-12-dUTP for 1 h and nuclei were counterstained with DAPI. Slides were observed under an AxioPlan2 microscope (Carl Zeiss, Jena, Germany).

### 4.9. Western Blot Analysis

HTR-8/SVneo and JEG-3 cell pellets were lysed using RIPA lysis buffer (1% NP-40, 0.1% SDS, 0.5% sodium deoxycholate, 150 mM NaCl and 50 mM Tris–HCl) containing protease and phosphatase inhibitors. Total protein concentrations were assessed using the Pierce™ Micro BCA™ Protein-Assay (Thermo Scientific). EV fractions (5 µg) were mixed with non-reducing Laemmli loading buffer (375 mM Tris.HCl, 9% SDS, 50% glycerol, 0.03% bromophenol blue) for analysis of EV markers. Cell lysates (30 µg) were mixed with reducing Laemmli buffer (adding 9% v/v ß-mercaptoethanol) for remaining proteins. Protein extracts were loaded on a 12 % precast gel SERVAGel™ (SERVA Electrophoresis GmbH, Heidelberg Germany), and resolved proteins were transferred to a nitrocellulose membrane (Hybond-P; GE Healthcare, Freiburg, Germany). Non-specific binding sites were blocked by incubation with TBST containing 5% (w/v) non-fat dried milk for 1 h at room temperature. Membranes were immunoblotted with specific primary antibodies overnight at 4 °C, followed by 1 h incubation at room temperature with the respective HRP-conjugated secondary antibody. The following primary monoclonal antibodies (rabbit) were purchased from Cell Signaling Technology Inc. (Danvers, MA, USA) and diluted 1:1000: anti-PTEN (catalogue number 9552S), anti-PDCD4 (catalogue number 9535S), anti-AKT (catalogue number 4685S), anti-phospho-AKT (p-AKT; catalogue number 4060S), anti-cleaved-caspase-3 (catalogue number 9664S) and anti-beta-actin (catalogue number 8457S). Anti-rabbit-HRP was diluted 1:10,000 (catalogue number 7074P2). For EV marker detection, the following primary antibodies were used at a 1:500 dilution: mouse anti-human CD63 (Thermo Scientific, catalogue number 10628D) and rabbit anti-human GAPDH (Cell Signaling, catalogue number 2118S); or 1:300 dilution: rabbit anti-human ALIX (Cell Signaling, catalogue number 928805) and mouse anti-human TSG101 (Abcam, catalogue number ab83). For detection, the respective anti-rabbit-HRP or anti-mouse-HRP (Cell Signaling, catalogue number 7076P2) secondary antibodies were diluted 1:3000. Blots were developed using an enhanced chemiluminescence (ECL) detection kit (Millipore, Schwalbach, Germany). Intensity of bands was analyzed and quantified by a MF-ChemiBis 3.2 gel documentation system with Totallab TL100 software version 2006 (Biostep GmbH, Jahnsdorf, Germany) and normalized to beta-actin.

### 4.10. AKT Activation in HTR-8/SVneo Cells

Transfected HTR-8/SVneo cells were serum-starved for 2 h before stimulation with 1 ng/µL EGF (Merck, Darmstadt, Germany) for 5 min. Cells were harvested and protein expression was quantified as described before in the “Western blot analysis” section. 

### 4.11. Cellular Uptake of Tophoblast-Derived EVs

EVs resuspended in 500 µL PBS were labelled with 2 µM PKH67 dye (Sigma-Aldrich, Munich, Germany) for 5 min. For the PKH67-PBS control, an equivalent volume of PBS was used as starting material. The reaction was stopped by adding an equal volume of 1% BSA. Pellets containing labelled EVs were washed twice with PBS by centrifugation at 100,000× *g* for 70 min. Recipient cells were seeded into 6-well plates and incubated with 125 ng/mL PKH67-labelled EV for 24 h in 2 mL medium containing ED-FBS. After washing, fluorescence in recipient cells was detected with an Accuri C6 plus cytometer (Becton Dickinson) and analyzed by using the manufacturer’s software.

For confocal examinations, 20,000 cells were seeded in Ibidi Chambered Coverslips (HTR-8/SVneo and JEG-3) or in 48-well plates (Jurkat T and NK92 cells) in 500 µL ED-FBS medium and treated with 125 ng/mL PKH67 stained EVs (lEV_PKH67_ and sEV_PKH67_) or an equivalent volume of PBS_PKH67_. After 24 h, the cultures were washed 3 times with cooled PBS and fixed with 2% formaldehyde for 10 min at room temperature; 1 µg/mL DAPI (catalogue number D9542-5MG; Sigma Aldrich) staining was used to visualize nuclei. Cellular uptake of trophoblast-derived EVs was observed and recorded using a Zeiss LSM 710 confocal laser microscope with an oil-immersion Plan-Apochromat 63x NA 1.4 objective (Carl Zeiss Microscopy GmbH) with 405 nm (DAPI) and 488 nm (PKH67) lasers. The 3D data reconstruction was made using a maximum intensity projection algorithm implemented in the Zeiss ZEN lite blue 2.5 software (Carl Zeiss Microscopy GmbH). This 3D visualization method is based on the projection of the most intense voxels along rays orthogonal to the projection plane. This method allows to present the localization of EVs inside the cell relative to other structures, such as the nucleus.

### 4.12. Bioinformatic Prediction of miR-519d-3p Target Genes 

Potential miR-519d-3p targets were selected from the bioinformatics platforms Microrna (https://www.microrna.org), PITA (http://genie.weizmann.ac.il) and Diana Micro-T V3.0 (http://diana.cslab.ece.ntua.gr/microT). Targets were confirmed by Western blotting as described above. 

### 4.13. Statistical Analysis

Each experiment was repeated independently at least three times. Values were expressed as means ± standard deviation (SD). Statistical analyses were performed by two-tailed Student’s *t* test, 2-way ANOVA test or 1-way ANOVA with a Bonferroni multiple comparisons test, as described in the figure legends. * *p* value < 0.05, ** *p* value < 0.01, *** *p* value < 0.001.

## Supplementary Materials

Supplementary materials can be found at https://www.mdpi.com/1422-0067/21/10/3458/s1. Supplementary Figure S1. Movie 1. Visualization of autologous internalized lEV_PKH67_ after 24 h by HTR-8/SVneo cells. HTR-8/SVneo cells turn around X. HTR-8-SVneo + lEV (Turn around X).wmv. Supplementary Figure S2. Movie 2. Treatment with PBS_PKH67_ of HTR-8/SVneo cells. HTR-8/SVneo cells turn around X. HTR-8-SVneo + PBS-PKH67 (Turn around X).wmv. Supplementary Figure S3. Movie 3. Visualization of autologous internalized sEV_PKH67_ after 24 h by HTR-8/SVneo cells. HTR-8/SVneo cells turn around X. HTR-8-SVneo + sEV (Turn around X).wmv. Supplementary Figure S4. Movie 4. Visualization of autologous internalized lEV_PKH67_ after 24 h by JEG-3 cells. JEG-3cells turn around X. JEG-3+ lEV (Turn around X).wmv. Supplementary Figure S5. Movie 5. Visualization of JEG-3 cells treated with PBS_PKH67_ of. JEG-3 cells turn around X. JEG-3 + PBS-PKH67 (Turn around X).wmv. Supplementary Figure S6. Movie 6. Visualization of autologous internalized sEV_PKH67_ after 24 h by JEG-3 cells. JEG-3 cells turn around X. JEG-3 + sEV (Turn around X).wmv. Supplementary Figure S7. Movie 7. Visualization of heterologous internalized lEV_PKH67_ after 24 h Jurkat T cells. Jurkat T cells turn around X. Jurkat + lEV (Turn around X).wmv. Supplementary Figure S8. Movie 8. Visualization of Jurkat T cells treated with PBS_PKH67_. Jurkat T cells turn around X. Jurkat + PBS-PKH67 (Turn around X).wmv. Supplementary Figure S9. Movie 9. Visualization of heterologous internalized sEV_PKH67_ after 24 h by Jurkat T cells. Jurkat T cells turn around X. Jurkat + sEV (Turn around X).wmv. Supplementary Figure S10. Movie 10. Visualization of heterologous internalized lEV_PKH67_ after 24 h by NK92 cells. NK92 cells turn around X. NK92+ lEV (Turn around X).wmv. Supplementary Figure S11. Movie 11. Visualization of NK92 cells treated with PBS_PKH67_. NK92 cells turn around X. NK92 + PBS-PKH67 (Turn around X).wmv. Supplementary Figure S12. Movie 12. Visualization of heterologous internalized sEV_PKH67_ after 24 h by NK92 cells. NK92 cells turn around X. NK92+ sEV (Turn around X).wmv.

## Abbreviations

ED-FBS	exosome depleted fetal bovine serum
EVs	extracellular vesicles
NTC	non-treated cells
sEV	small EV
lEV	large EV
sEV_miR-519d_	sEV derived from miR-519d mimic transfected cells
sEV_SCR_	sEV derived from non-genomic SCR mimic transfected cells
sEV_CTR_	sEV derived from non-transfected cells
lEV_miR-519d_	lEV derived from miR-519d mimic transfected cells
lEV_SCR_	lEV derived from non-genomic SCR mimic transfected cells
lEV_CTR_	lEV derived from non-transfected cells
sEV_PKH67_	sEV stained with PKH67
lEV_PKH67_	lEV stained with PKH67
PBS_PKH67_	pellet of PBS stained with PKH67 following the same protocol as for EV_PKH67_

## References

[1] Anin S.A., Vince G., Quenby S. (2004). Trophoblast invasion. Hum. Fertil..

[2] Knofler M., Pollheimer J. (2012). IFPA Award in Placentology lecture: Molecular regulation of human trophoblast invasion. Placenta.

[3] Helige C., Ahammer H., Moser G., Hammer A., Dohr G., Huppertz B., Sedlmayr P. (2014). Distribution of decidual natural killer cells and macrophages in the neighbourhood of the trophoblast invasion front: A quantitative evaluation. Hum. Reprod..

[4] Liu S., Diao L., Huang C., Li Y., Zeng Y., Kwak-Kim J.Y.H. (2017). The role of decidual immune cells on human pregnancy. J. Reprod. Immunol..

[5] Lash G.E., Ernerudh J. (2015). Decidual cytokines and pregnancy complications: Focus on spontaneous miscarriage. J. Reprod. Immunol..

[6] Bidarimath M., Khalaj K., Wessels J.M., Tayade C. (2014). MicroRNAs, immune cells and pregnancy. Cell. Mol. Immunol..

[7] Robertson S.A., Zhang B., Chan H., Sharkey D.J., Barry S.C., Fullston T., Schjenken J.E. (2017). MicroRNA regulation of immune events at conception. Mol. Reprod. Dev..

[8] Morales Prieto D.M., Markert U.R. (2011). MicroRNAs in pregnancy. J. Reprod. Immunol..

[9] Chaiwangyen W., Ospina-Prieto S., Photini S.M., Schleussner E., Markert U.R., Morales-Prieto D.M. (2015). Dissimilar microRNA-21 functions and targets in trophoblastic cell lines of different origin. Int. J. Biochem. Cell Biol..

[10] Morales-Prieto D.M., Ospina-Prieto S., Schmidt A., Chaiwangyen W., Markert U.R. (2014). Elsevier Trophoblast Research Award Lecture: Origin, evolution and future of placenta miRNAs. Placenta.

[11] Slezak-Prochazka I., Durmus S., Kroesen B.J., van den Berg A. (2010). MicroRNAs, macrocontrol: Regulation of miRNA processing. RNA.

[12] Bartel D.P. (2004). MicroRNAs: Genomics, biogenesis, mechanism, and function. Cell.

[13] Morales-Prieto D.M., Ospina-Prieto S., Chaiwangyen W., Schoenleben M., Markert U.R. (2013). Pregnancy-associated miRNA-clusters. J. Reprod. Immunol..

[14] Morales-Prieto D.M., Chaiwangyen W., Ospina-Prieto S., Schneider U., Herrmann J., Gruhn B., Markert U.R. (2012). MicroRNA expression profiles of trophoblastic cells. Placenta.

[15] Higashijima A., Miura K., Mishima H., Kinoshita A., Jo O., Abe S., Hasegawa Y., Miura S., Yamasaki K., Yoshida A. (2013). Characterization of placenta-specific microRNAs in fetal growth restriction pregnancy. Prenat. Diagn..

[16] Gilad S., Meiri E., Yogev Y., Benjamin S., Lebanony D., Yerushalmi N., Benjamin H., Kushnir M., Cholakh H., Melamed N. (2008). Serum microRNAs are promising novel biomarkers. PLoS ONE.

[17] Xie L., Sadovsky Y. (2016). The function of miR-519d in cell migration, invasion, and proliferation suggests a role in early placentation. Placenta.

[18] Donker R.B., Mouillet J.F., Chu T., Hubel C.A., Stolz D.B., Morelli A.E., Sadovsky Y. (2012). The expression profile of C19MC microRNAs in primary human trophoblast cells and exosomes. Mol. Hum. Reprod..

[19] Ospina-Prieto S., Chaiwangyen W., Herrmann J., Groten T., Schleussner E., Markert U.R., Morales-Prieto D.M. (2016). MicroRNA-141 is upregulated in preeclamptic placentae and regulates trophoblast invasion and intercellular communication. Transl. Res..

[20] Gaus G., Funayama H., Huppertz B., Kaufmann P., Frank H.G. (1997). Parent cells for trophoblast hybridization I: Isolation of extravillous trophoblast cells from human term chorion laeve. Placenta.

[21] Graham C.H., Hawley T.S., Hawley R.G., MacDougall J.R., Kerbel R.S., Khoo N., Lala P.K. (1993). Establishment and characterization of first trimester human trophoblast cells with extended lifespan. Exp. Cell Res..

[22] Bilban M., Tauber S., Haslinger P., Pollheimer J., Saleh L., Pehamberger H., Wagner O., Knofler M. (2010). Trophoblast invasion: Assessment of cellular models using gene expression signatures. Placenta.

[23] Novakovic B., Gordon L., Wong N.C., Moffett A., Manuelpillai U., Craig J.M., Sharkey A., Saffery R. (2011). Wide-ranging DNA methylation differences of primary trophoblast cell populations and derived cell lines: Implications and opportunities for understanding trophoblast function. Mol. Hum. Reprod..

[24] Théry C., Witwer K.W., Aikawa E., Alcaraz M.J., Anderson J.D., Andriantsitohaina R., Antoniou A., Arab T., Archer F., Atkin-Smith G.K. (2018). Minimal information for studies of extracellular vesicles 2018 (MISEV2018): A position statement of the International Society for Extracellular Vesicles and update of the MISEV2014 guidelines. J. Extracell. Vesicles.

[25] Nguyen P.N., Huang C.J., Sugii S., Cheong S.K., Choo K.B. (2017). Selective activation of miRNAs of the primate-specific chromosome 19 miRNA cluster (C19MC) in cancer and stem cells and possible contribution to regulation of apoptosis. J. Biomed. Sci..

[26] Liang Y., Ridzon D., Wong L., Chen C. (2007). Characterization of microRNA expression profiles in normal human tissues. BMC Genom..

[27] Fu G., Brkic J., Hayder H., Peng C. (2013). MicroRNAs in Human Placental Development and Pregnancy Complications. Int. J. Mol. Sci..

[28] Xie L., Mouillet J.F., Chu T., Parks W.T., Sadovsky E., Knofler M., Sadovsky Y. (2014). C19MC microRNAs regulate the migration of human trophoblasts. Endocrinology.

[29] Kim K.M., Abdelmohsen K., Mustapic M., Kapogiannis D., Gorospe M. (2017). RNA in extracellular vesicles. Wiley Interdiscip. Rev. RNA.

[30] Gyorgy B., Hung M.E., Breakefield X.O., Leonard J.N. (2015). Therapeutic applications of extracellular vesicles: Clinical promise and open questions. Annu. Rev. Pharmacol. Toxicol..

[31] Corcoran C., Friel A.M., Duffy M.J., Crown J., O’Driscoll L. (2011). Intracellular and extracellular microRNAs in breast cancer. Clin. Chem..

[32] Fornari F., Milazzo M., Chieco P., Negrini M., Marasco E., Capranico G., Mantovani V., Marinello J., Sabbioni S., Callegari E. (2012). In hepatocellular carcinoma miR-519d is up-regulated by p53 and DNA hypomethylation and targets CDKN1A/p21, PTEN, AKT3 and TIMP2. J. Pathol..

[33] Zhao Z., Zhao Q., Warrick J., Lockwood C.M., Woodworth A., Moley K.H., Gronowski A.M. (2012). Circulating microRNA miR-323-3p as a biomarker of ectopic pregnancy. Clin. Chem..

[34] Hromadnikova I., Kotlabova K., Ondrackova M., Pirkova P., Kestlerova A., Novotna V., Hympanova L., Krofta L. (2015). Expression Profile of C19MC microRNAs in Placental Tissue in Pregnancy-Related Complications. DNA Cell Biol..

[35] Ding J., Huang F., Wu G., Han T., Xu F., Weng D., Wu C., Zhang X., Yao Y., Zhu X. (2015). MiR-519d-3p suppresses invasion and migration of trophoblast cells via targeting MMP-2. PLoS ONE.

[36] Zhou J.Y., Zheng S.R., Liu J., Shi R., Yu H.L., Wei M. (2016). MiR-519d facilitates the progression and metastasis of cervical cancer through direct targeting Smad7. Cancer Cell Int..

[37] Pang Y., Mao H., Shen L., Zhao Z., Liu R., Liu P. (2014). MiR-519d represses ovarian cancer cell proliferation and enhances cisplatin-mediated cytotoxicity in vitro by targeting XIAP. Onco Targets Ther..

[38] Morales-Prieto D.M., Barth E., Murrieta-Coxca J.M., Favaro R.R., Gutiérrez-Samudio R.N., Chaiwangyen W., Ospina-Prieto S., Gruhn B., Schleußner E., Marz M. (2019). Identification of miRNAs and associated pathways regulated by Leukemia Inhibitory Factor in trophoblastic cell lines. Placenta.

[39] van der Pol E., Coumans F.A., Grootemaat A.E., Gardiner C., Sargent I.L., Harrison P., Sturk A., van Leeuwen T.G., Nieuwland R. (2014). Particle size distribution of exosomes and microvesicles determined by transmission electron microscopy, flow cytometry, nanoparticle tracking analysis, and resistive pulse sensing. J. Thromb. Haemost..

[40] van Niel G., Charrin S., Simoes S., Romao M., Rochin L., Saftig P., Marks M.S., Rubinstein E., Raposo G. (2011). The tetraspanin CD63 regulates ESCRT-independent and -dependent endosomal sorting during melanogenesis. Dev. Cell.

[41] Raiborg C., Stenmark H. (2009). The ESCRT machinery in endosomal sorting of ubiquitylated membrane proteins. Nature.

[42] Ouyang Y., Bayer A., Chu T., Tyurin V.A., Kagan V.E., Morelli A.E., Coyne C.B., Sadovsky Y. (2016). Isolation of human trophoblastic extracellular vesicles and characterization of their cargo and antiviral activity. Placenta.

[43] PrabhuDas M., Bonney E., Caron K., Dey S., Erlebacher A., Fazleabas A., Fisher S., Golos T., Matzuk M., McCune J.M. (2015). Immune mechanisms at the maternal-fetal interface: Perspectives and challenges. Nat. Immunol..

[44] Warning J.C., McCracken S.A., Morris J.M. (2011). A balancing act: Mechanisms by which the fetus avoids rejection by the maternal immune system. Reproduction.

[45] Park S.Y., Yun S., Ryu B.J., Han A.R., Lee S.K. (2017). Trophoblasts regulate natural killer cells via control of interleukin-15 receptor signaling. Am. J. Reprod. Immunol..

[46] Tao Y., Li Y.H., Piao H.L., Zhou W.J., Zhang D., Fu Q., Wang S.C., Li D.J., Du M.R. (2015). CD56(bright)CD25+ NK cells are preferentially recruited to the maternal/fetal interface in early human pregnancy. Cell. Mol. Immunol..

[47] Rajagopalan S. (2014). HLA-G-mediated NK cell senescence promotes vascular remodeling: Implications for reproduction. Cell. Mol. Immunol..

[48] Tannetta D., Dragovic R., Alyahyaei Z., Southcombe J. (2014). Extracellular vesicles and reproduction-promotion of successful pregnancy. Cell. Mol. Immunol..

[49] Mulcahy L.A., Pink R.C., Carter D.R. (2014). Routes and mechanisms of extracellular vesicle uptake. J. Extracell. Vesicles.

[50] Kurian N.K., Modi D. (2019). Extracellular vesicle mediated embryo-endometrial cross talk during implantation and in pregnancy. J. Assist. Reprod. Genet..

[51] Alam S.M.K., Jasti S., Kshirsagar S.K., Tannetta D.S., Dragovic R.A., Redman C.W., Sargent I.L., Hodes H.C., Nauser T.L., Fortes T. (2018). Trophoblast Glycoprotein (TPGB/5T4) in Human Placenta: Expression, Regulation, and Presence in Extracellular Microvesicles and Exosomes. Reprod. Sci..

[52] Frängsmyr L., Baranov V., Nagaeva O., Stendahl U., Kjellberg L., Mincheva-Nilsson L. (2005). Cytoplasmic microvesicular form of Fas ligand in human early placenta: Switching the tissue immune privilege hypothesis from cellular to vesicular level. Mol. Hum. Reprod..

[53] Stenqvist A.C., Nagaeva O., Baranov V., Mincheva-Nilsson L. (2013). Exosomes secreted by human placenta carry functional Fas ligand and TRAIL molecules and convey apoptosis in activated immune cells, suggesting exosome-mediated immune privilege of the fetus. J. Immunol..

[54] Giacomini E., Alleva E., Fornelli G., Quartucci A., Privitera L., Vanni V.S., Viganò P. (2019). Embryonic extracellular vesicles as informers to the immune cells at the maternal-fetal interface. Clin. Exp. Immunol..

[55] Tong M., Abrahams V.M., Chamley L.W. (2018). Immunological effects of placental extracellular vesicles. Immunol. Cell Biol..

